# Incidence and management of overdrainage in pediatric hydrocephalus patients exclusively treated with modern gravitational VP shunt valves

**DOI:** 10.1007/s00381-026-07185-0

**Published:** 2026-02-27

**Authors:** B. Younes, F. Knerlich-Lukoschus, H. C. Bock

**Affiliations:** 1 Department of Neurosurgery, University Medical Center Göttingen, Göttingen University Hospital, Georg-August-University of Göttingen, Robert-Koch-Straße 40, 37075 Göttingen, Germany; 2 Department of Neurosurgery, Division of Pediatric Neurosurgery, University Medical Center Göttingen, Robert-Koch-Str. 40, 37075 Göttingen, Germany

**Keywords:** Overdrainage, Hyperdrainage, Excessive CSF drainage, Overshunting, CSF shunting, VP shunting, Hydrocephalus

## Abstract

**Objective:**

Cerebrospinal fluid (CSF)-overdrainage (OD) may occur in short- or long-time shunt treatment with a variety of clinical symptoms or radiographic findings ranging from mild postural headaches to chronic hygroma or even acute subdural hematoma. Shunt-valve pressure level adjustability and prevention of the siphoning effect with anti-siphon devices and gravitational units have fundamental influence on the current standards of modern CSF-valve technology. However, OD may also occur despite the upfront use of those prophylactic technologies. The aim of this study is to determine incidence and clinical management of OD in pediatric hydrocephalus patients treated with modern gravitational valve shunts.

**Methods:**

Using our institutional pediatric Hydrocephalus and Shunt Registry providing more than 700 individual shunt histories of varying hydrocephalus etiology, we analyzed the clinical course of all patients who were equipped with gravitational valves right from the start. Initial valve-type and pressure level settings as well as all subsequent pressure level adjustments or surgical valve-type alterations were analyzed corresponding to clinical and neuroimaging findings. The inclusion criteria were complete institutional ventricular CSF-shunt history and follow-up with initial insertion of a gravitational shunt valve. Time-to-event analyses used time from primary VP shunt implantation to first OD event; patients without OD were censored at last follow-up.

**Results:**

Two hundred twenty-three patients met the inclusion criteria with a mean follow-up of 5.6 ± 2.1 years. Patients were initially equipped with the M.blue valve in 40 patients (18%) or the miniNAV/proSA valve in 144 patients (65%). During the entire follow-up, 78 patients (35%) developed clinical and/or radiographic signs of OD, which were effectively manageable by pressure level adjustments alone in 81% and surgical intervention in 19% of the cases. The frequency of valve adjustments averaged 1.7 per patient. The mean initial pressure settings were 5.17 cmH₂O for differential pressure valve (DPV), 19.54 cmH₂O for gravitational unit (GAV), and 24.71 cmH₂O for upright pressure. At the latest follow-up, the mean pressure settings had increased to 5.44 cmH₂O for DPV, 21.69 cmH₂O for GAV, and 27.13 cmH₂O for upright pressure. In patients with the M.blue valve, 16 out of 40 (36%) experienced OD. For the miniNAV/proSA valve, 56 out of 144 patients (39%) showed signs of OD. In comparison, the proGAV valve had the lowest OD occurrence, with only 6 out of 39 patients (15%) affected. Temporary shunt ligation was performed in 2 patients, while an additional subdural hematoma (SDH) or hygroma drainage was needed in another 2 patients.

**Conclusion:**

Long-term follow-up monitoring of shunt-treated pediatric patients reveals a not negligible incidence of OD even though the shunt system is already equipped with a preventive gravitational unit. Pressure level adjustments can effectively counteract corresponding clinical symptoms and radiographic signs. The proGAV valve demonstrated significantly longer adjustment-free survival with lower rates of OD-related interventions, indicating a potential advantage in reducing long-term complications.

## Introduction

The use of cerebrospinal fluid (CSF) shunts in the early 1950 s was revolutionary in neurosurgery, reducing mortality rates in patients with hydrocephalus from approximately 70% to less than 1% [1]. The first generation of ventriculoperitoneal shunts (VPS) was simple, open systems without valves [2]. The 1960 s saw the introduction of pressure-sensitive valves, which allowed CSF to flow only when intracranial pressure exceeded a certain threshold. These early valves were fixed-pressure systems and not adjustable [3]. The term “overdrainage” was first introduced in the literature in 1968 and was defined as a complication due to excessive therapeutic drainage of the CSF [1,. The reported incidence of OD ranges widely, from 2 to 71%. This significant variation is largely attributed to inconsistent diagnostic criteria. Clinical symptoms and treatment approaches for OD can differ between adults and children and may also vary among children of different age groups [4–6].

**Fig. 1:**
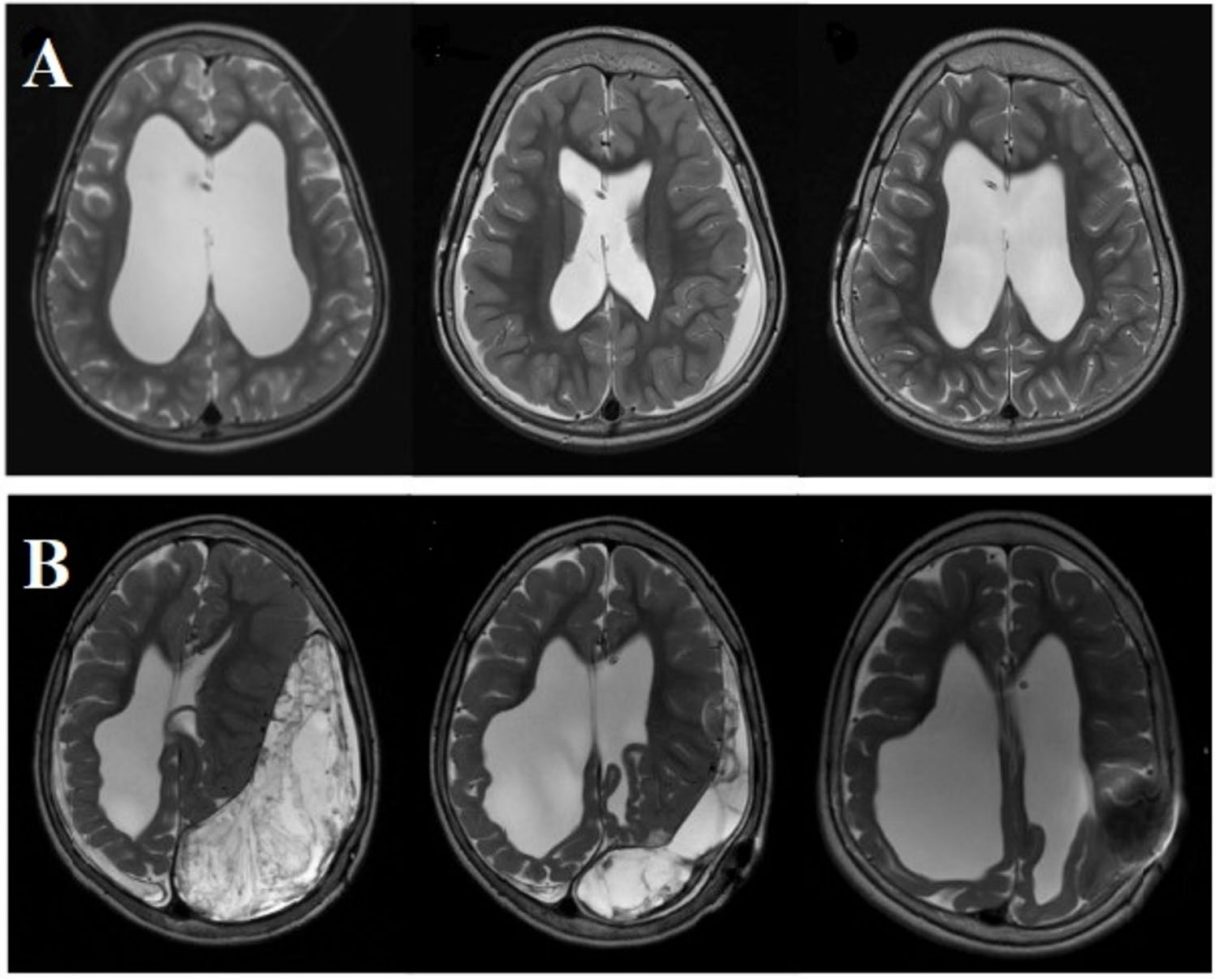
Follow-up MRI scans of two different pediatric patients with hydrocephalus who were surgically treated with VPs. Both patients presented with clinical and radiological signs of overdrainage. **A** Showed hyperostosis and subdural hematoma, while (**B**) exhibited a subdural hygroma. Both cases were successfully managed through shunt pressure level adjustments

However, challenges arose, revealing that different pathologies required tailored shunt settings depending on the patient’s age and etiology. This led to the development of programmable valves in the 1980 s and, later, to anti-siphon devices, allowing for improved control of CSF drainage [1, 7]. Despite these advancements, issues with overdrainage and underdrainage continued to arise. Among the forces affecting drainage, intraventricular pressure drives CSF drainage, while the resistance within the shunt system and the pressure in the peritoneal cavity act as counterforces [8, 9]. Additionally, in a lying patient, the hydrostatic difference between the cerebral ventricles and the abdominal cavity is approximately zero. However, in a standing patient, hydrostatic forces of 20–60 cmH₂O come into effect, significantly impacting CSF drainage [10, 11]. Cerebrospinal fluid OD may become apparent during short- or long-term shunt treatment. In children, OD rarely leads to acute symptoms but often results in chronic anatomical changes, which may only be identified after several years of shunting. Clinical symptoms and/or radiographic findings can range from mild postural headaches to chronic hygroma due to the tearing of bridging veins, shunt dysfunction because of slit ventricle syndrome, premature closure of cranial sutures (in infants), low intracranial pressure (ICP) syndrome, or even acute subdural hematoma (Fig. 1) [12–14]. Other common clinical findings include drowsiness, visual disturbances, nausea, vomiting, paleness, feeding difficulties, preference for a horizontal position, slowed head growth, difficulty concentrating, reduced performance, and developmental delays [12–14].

Shunt-valve pressure level adjustability and prevention of the siphoning effect with anti-siphon devices and gravitational units had fundamental influence on the current standards of modern CSF-valve technology. Even though OD may also occur despite the upfront use of those prophylactic technologies [15, 16], the aim of this study is to determine incidence and clinical management of OD in pediatric hydrocephalus patients treated with modern gravitational valve shunts.

## Materials and methods

### Patients

This study is a retrospective analysis using the Pediatric Hydrocephalus and Shunt Registry at University Hospital Göttingen between 2012 and 2024, which provides data of 723 individual shunt histories across various hydrocephalus etiologies. The decision for VPS was based on repeated cranial ultrasound examinations or MRI scans regarding to ventricular enlargement, increasing head circumference, bulging fontanel, or other clinical signs of raised intracranial pressure. The inclusion criteria were a complete institutional ventricular CSF shunt history and follow-up with the initial insertion of a gravitational shunt valve. We excluded all patients who were not operated on at our institution, all non-ventriculo-peritoneal shunts, patients who did not receive gravitational valve ventriculoperitoneal shunts from the outset, and cases with incomplete or missing data and follow-up.

### Postoperative management

We analyzed the clinical course of all patients who were equipped with gravitational valves from the outset. Initial valve type and pressure level settings, as well as all subsequent pressure adjustments or surgical changes in valve type, were assessed in relation to clinical and neuroimaging findings. The clinical follow-up protocol includes comprehensive evaluations to ensure optimal shunt function and patient health. Key assessments involve monitoring patient complaints, head circumference (especially in infants), developmental milestones and papilledema. Physical exams, including reservoir palpation, help detect under- or OD. Pressure levels are reviewed and adjusted as needed based on clinical findings, providing a proactive approach to managing potential complications.

### Overdrainage definition and management

OD was defined as any patient presenting with clinical symptoms alone (such as postural headache that worsens when sitting or standing, nausea/vomiting, dizziness, sunken fontanelle, slowed head growth, or other signs of low intracranial pressure), neuroimaging findings alone (including slit ventricles, thickened and contrast-enhancing dura, hyperostosis, subdural hygroma or hematoma, or early suture fusion), or a combination of both clinical and radiological signs. Regarding OD, routine long-term follow-up includes monitoring head circumference to ensure appropriate growth, reservoir pressure testing, and funduscopic examination twice yearly. Event time was defined as the earliest date on which OD was first documented in the medical record, based on clinical assessment and/or neuroimaging meeting the OD definition. If OD is suspected, cranial imaging is updated to confirm the diagnosis and to exclude associated intracranial complications, such as hygroma or subdural hematoma. When slit ventricles or other imaging findings correlate with clinical symptoms, the valve pressure level is adjusted stepwise in increments of 2–5 cm H₂O. In acute cases, intravenous fluid administration and bed rest may be indicated. Four to 6 weeks after valve adjustment, additional fast-sequence MRI and clinical follow-up are performed.

### Follow-up assessments

Post-operative X-rays (shunt series) were performed to confirm correct implant positioning, adjustment, and integrity of the implant. Regular clinical follow-ups were scheduled perioperatively, on day 10 post-surgery, at 3, 6, and 12 months after surgery, and then annually. For patients under 12 months, ultrasound was routinely performed at each follow-up. MRI imaging was scheduled at 3 and 12 months post-surgery, with additional MRIs as clinically indicated.

### Statistical methods

The analysis included descriptive statistics (mean and range) and Kaplan–Meier survival analysis used to summarize continuous variables such as age, follow-up duration, and pressure settings. An Excel database (Microsoft Corp) and exported to SPSS or statistical analysis, which was performed using IBM SPSS Statistics Version 27.0 (IBM Corp, Released 2016, IBM SPSS Statistics for Windows, Version 27.0, Armonk, NY, USA).

### Time-to-event analyses

Time zero was the date of primary VP shunt implantation. The event for Cox regression was the first occurrence of OD as defined above (clinical OD, imaging OD, or both). Patients without OD were censored at the date of last documented clinical/imaging follow-up. For Kaplan–Meier analyses of OD-adjustment–free survival, the event was the first OD-related valve pressure increase; patients without OD-related adjustments were censored at last follow-up. Time was analyzed in months. For patients with multiple OD episodes, only the first OD event was used for time-to-event analyses.

### Ethics statement

Ethical approval was secured (ethical commission of University Hospital Göttingen, application number: 12/9/17), aligning with the 1964 Declaration of Helsinki and its amendments. All procedures adhered to local and institutional laws and data protection regulations.

## Results

**Fig. 2:**
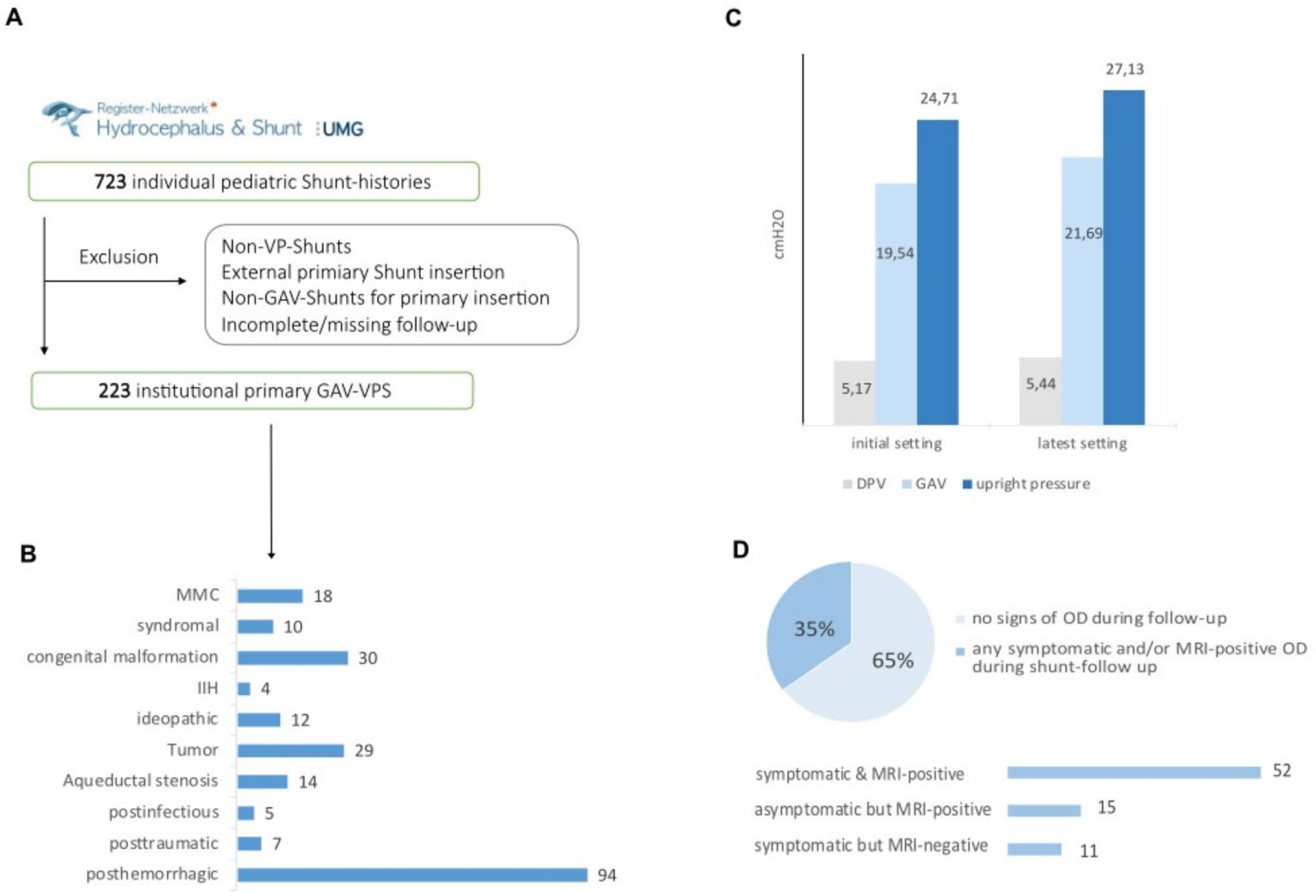
**A** The flowchart shows the search mechanisms. **B** Different causes of pediatric hydrocephalus in our cohort. **C** The mean initial and latest pressure settings for differential pressure valve (DPV), GAV, and upright pressure. **D** The relationship between symptoms and MRI findings

**Fig. 3:**
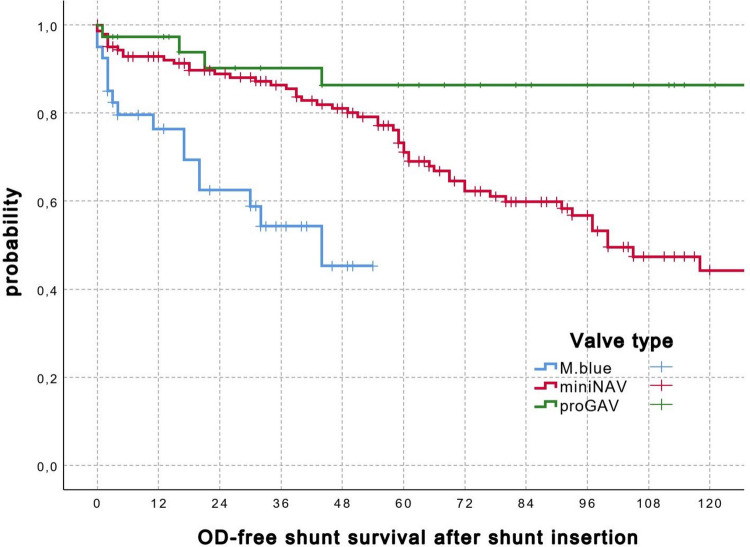
Kaplan–Meier curves showing OD-related valve–adjustment–free survival for the three valve types (miniNAV/proSA, proGAV, and M.blue). A significant difference between survival curves was observed (log-rank *p* < 0.001). Time is in months after shunt implantation

The initial data collection from the Pediatric Hydrocephalus and Shunt Registry at University Hospital Göttingen included 723 patients with a primary diagnosis of pediatric hydrocephalus and shunt interventions. After applying the predefined exclusion criteria, a cohort of 223 pediatric patients with hydrocephalus, initially treated at our institute with gravitational valve ventriculoperitoneal shunts, remained for analysis (Fig. 2A). The mean follow-up was 5.6 ± 2.1 years. The most common cause of hydrocephalus was post-hemorrhagic, observed in 94 patients (42%), followed by congenital malformation in 30 patients (13%). Figure 2B shows the most frequent causes of hydrocephalus in our cohort. The mean age at surgery was 2.6 years. The shunt insertion surgeries were conducted by experienced consultant neurosurgeons, with the average duration of the procedures recorded at 47.4 ± 16 min. The most commonly used GAV valve was the miniNAV/proSA (fixed DPV + programmable gravitational unit) in 144 patients (65%), followed by the M.blue (programmable DPV + fixed gravitational unit) in 40 patients (18%). At the last follow-up, the number of patients with miniNAV/proSA decreased to 115 (52%), while those with M.blue increased to 60 (27%). The types of valves used are shown in Table 1. The transition patterns of OD-related outcomes across valve types are summarized in Table 2. Throughout the entire follow-up period, 78 patients (35%) developed clinical and/or radiographic signs of OD. These signs were effectively managed through pressure level adjustments alone in 81% of these cases. The frequency of valve adjustments, averaging 1.7 per patient (with a range of 0 to 15), highlights the adaptability of gravitational valve ventriculoperitoneal shunts but also suggests a need for ongoing monitoring and occasional adjustments to optimize intracranial pressure regulation. The need for shunt revision surgeries averaging 0.98 per patient (ranging from 0 to 11) shows that while many patients do not require frequent surgical interventions, some may experience complex clinical courses that necessitate repeated revisions. The mean initial pressure settings for differential pressure valve (DPV), GAV, and upright pressure were analyzed. Initially, the average pressure settings were 5.17 cmH₂O for DPV, 19.54 cmH₂O for GAV, and 24.71 cmH₂O for upright pressure. At the latest follow-up, the mean pressure settings had increased to 5.44 cmH₂O for DPV, 21.69 cmH₂O for GAV, and 27.13 cmH₂O for upright pressure (Fig. 2C). Out of 78 patients (35%) with exclusive gravitational valve ventriculoperitoneal shunt treatment who showed signs of OD, GAV valve upregulation was required. Among these, 52 patients (67%) exhibited both symptomatic and radiographic signs of OD, while 11 patients (14%) showed only clinical symptoms of OD without radiographic signs and 15 patients (19%) showed radiographic signs without clinical symptoms (Fig. 2D). Among the different types of valves used, the occurrence of OD varied. In patients with the M.blue valve, 16 out of 40 (36%) experienced OD. For the miniNAV/proSA valve, 56 out of 144 patients (39%) showed signs of OD. In comparison, the proGAV valve had the lowest OD occurrence, with only 6 out of 39 patients (15%) affected. Out of the 78 patients with OD, 15 (19%) required additional revision surgery due to this complication. The mean time to revision surgery across these cases was 72 months, with a range of 0 to 140 months. In addition to standard procedures, several specific interventions were necessary among the patient cohort. Surgical valve exchange was required in 14 patients, representing 6% of the total population (Table 3). Temporary shunt ligation was performed in 2 patients, while an additional subdural hematoma (SDH) or hygroma drainage was needed in another 2 patients. One patient required a neuroendoscopic septostomy as a standalone intervention. Kaplan–Meier survival estimates demonstrated significant differences in OD-adjustment–free survival among the three valve types (miniNAV/proSA, proGAV, and M.blue). The proGAV valve showed the most favorable survival profile, with a mean OD-adjustment–free survival of 156 months (95% CI, 131.0–181.1). The miniNAV/proSA valve demonstrated intermediate performance with a mean survival of 99 months (95% CI 88.6–109.6). The M.blue valve exhibited the shortest adjustment-free survival, with a mean of 34 months (95% CI 27.0–41.5). The log-rank test revealed a highly significant difference between the survival curves (*χ*^2^ = 28.57, df = 2; *p* < 0.001) (Fig. 3).

**Table 1:** Shunt registry status considering valve type in pediatric patients with ventriculo-peritoneal shunts. Delta (Δ) represents the change in the number of valves between the initial insertion and the last follow-up

Valve type	Initially inserted valve	Last follow-up	Delta (Δ)
miniNAV/proSA	144 (65%)	115 (52%)	− 29
M.blue	40 (18%)	60 (27%)	+ 20
proGAV	32 (14%)	28 (13%)	− 4
proGAV 2.0	7 (3%)	11 (5%)	+ 4
M.blue plus	0	2 (1%)	+ 2
miniNAV/proSA + fixer SA	0	2 (1%)	+ 2
proGAV + proSA	0	2 (1%)	+ 2
Certas + M.blue	0	1	+ 1
M.blue + fixer SA	0	1	+ 1
proGAV 2.0 + M.blue	0	1	+ 1

**Table 2:** Transition matrix of overdrainage outcomes by valve type

Outcome measures	Valve type		
	miniNAV/proSA	proGAV	M.blue
Initially inserted (*N*)	144	39	40
Mean age at initial shunt insertion (years)	1.7	5.1	3.1
Mean initial upright pressure level (cmH2O)	23.9	25.9	26.4
Mean latest upright pressure level (cmH2O)	27.3	24.8	28.5
Mean number of valve adjustments	2.1	0.8	1.2
OD related pressure level enhancement, *N* (%)	56 (39%)	6 (15%)	16 (40%)
Time to OD-related pressure level increase (months)	54.2	57.6	12.8
Revision surgery due to OD, *N* (%)	12 (8%)	0 (0%)	3 (7%)

**Table 3:** VP shunt revisions for overdrainage: valve changes and temporal characteristics

No	Etiology	Initial valve	Initial pressure (cmH2O)	Primary insertion date	Revision date	Indication for revision	Revised valve	Revised pressure (cmH2O)
1	Posthemorrhagic	miniNAV/proSA	20	18 Feb 2011	27 Sep 2022	Nonadjustable valve	M.blue	25
2	Posthemorrhagic	miniNAV/proSA	25	31 Jul 2013	05 May 2021	Nonadjustable valve	M.blue	30
3	Posthemorrhagic	M.blue	21	28 Feb 2017	18 Jul 2017	Overdrainage despite high-pressure setting	proGAV + proSA	55
4	Aqueductal stenosis	miniNAV/proSA	25	11 Jan 2012	12 Jul 2023	Overdrainage despite high-pressure setting	M.blue	38
5	Posthemorrhagic	miniNAV/proSA	20	28 Sep 2012	10 Nov 2020	Overdrainage despite high-pressure setting	proGAV + proSA	65
6	Posthemorrhagic	miniNAV/proSA	20	27 Sep 2012	17 Aug 2022	Overdrainage despite high-pressure setting	M.blue	40
7	Posthemorrhagic	miniNAV/proSA	20	30 Oct 2012	29 May 2018	Overdrainage despite high-pressure setting	miniNAV/proSA + fixed SA	58
8	Idiopathic	miniNAV/proSA	25	12 Nov 2010	20 Oct 2021	Nonadjustable valve	M.blue	43
9	Posthemorrhagic	miniNAV/proSA	20	22 Feb 2011	10 Jul 2019	Overdrainage despite high-pressure setting	miniNAV/proSA + fixed SA	60
10	Posthemorrhagic	miniNAV/proSA	25	15 Aug 2018	29 Aug 2023	Overdrainage despite high-pressure setting	M.blue	35
11	Posthemorrhagic	miniNAV/proSA	25	22 Sep 2015	11 Nov 2020	Overdrainage with subdural hygroma	M.blue + fixed SA	70
12	Posthemorrhagic	miniNAV/proSA	20	16 Sep 2014	03 Mar 2020	Nonadjustable valve	M.blue	33
13	Posthemorrhagic	M.blue	25	10 Oct 2023	20 Dec 2023	Overdrainage despite high-pressure setting	M.blue	42
14	Congenital malformation	M.blue	25	16 Sep 2021	09 Feb 2022	Nonadjustable valve	proGAV 2.0 + fixed SA	33

### Minimally adjusted Cox analysis

In the minimally adjusted Cox proportional hazards model evaluating time from VP shunt implantation to first OD event, 223 patients were included and 78 OD events occurred. The model was adjusted for age at implantation, hydrocephalus etiology, baseline upright pressure (cmH₂O), and year of implantation. Year of implantation was independently associated with a higher hazard of OD (HR 1.28 per year; 95% CI 1.18–1.39; *p* < 0.001). In contrast, age at implantation (HR 1.05 per year; 95% CI 0.97–1.14; *p* = 0.249) and baseline upright pressure (HR 0.96 per 1 cmH₂O; 95% CI 0.89–1.04; *p* = 0.300) were not significantly associated with OD risk. Using posthemorrhagic hydrocephalus as the reference category, no etiology subgroup was significantly associated with OD in the adjusted model, including Aqueductal stenosis (HR 0.49; 95% CI 0.15–1.63; *p* = 0.245), spina bifida–associated hydrocephalus (HR 1.46; 95% CI 0.69–3.07; *p* = 0.320), tumor-associated hydrocephalus (HR 0.67; 95% CI 0.21–2.17; *p* = 0.501), congenital malformation (HR 0.80; 95% CI 0.38–1.69; *p* = 0.554), idiopathic hydrocephalus (HR 0.98; 95% CI 0.37–2.60; *p* = 0.965), idiopathic intracranial hypertension (HR 0.70; 95% CI 0.12–4.23; *p* = 0.699), postinfectious (HR 0.61; 95% CI 0.08–4.49; *p* = 0.625), posttraumatic (HR 2.40; 95% CI 0.68–8.50; *p* = 0.174) and syndromal hydrocephalus (HR 0.40; 95% CI 0.10–1.66; *p* = 0.206).

**Fig. 4:**
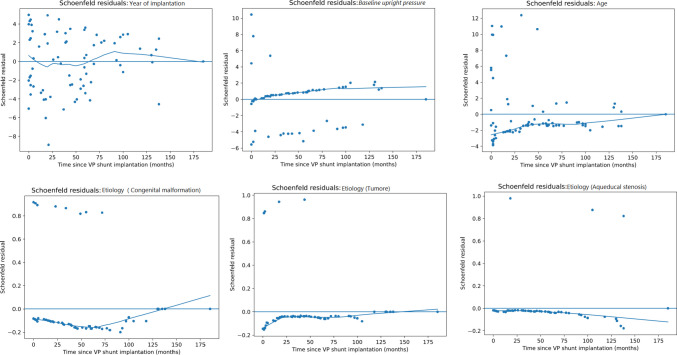
Schoenfeld residual plots for many covariates included in the Cox proportional hazards model. The absence of systematic trends over time indicates that the proportional hazards assumption is satisfied for all variables; age showed a borderline deviation

The proportional hazards assumption was evaluated using Schoenfeld residual diagnostics. The global test did not indicate violation (*χ*^2^ = 14.78; df = 12; *p* = 0.254). At the covariate level, age at implantation showed a borderline indication of non-proportionality (*p* = 0.045), whereas year of implantation (*p* = 0.734), baseline upright pressure (*p* = 0.179), and etiology indicators showed no evidence of time-varying effects (Fig. 4). Importantly, although year of implantation was a significant predictor of OD, Schoenfeld residuals supported that its effect remained proportional over follow-up.

To account for heterogeneous follow-up durations, OD incidence was reported as events per 100 patient-years. The overall incidence was 7.65 per 100 patient-years (78 events over 1019.25 patient-years; median follow-up 49 months [IQR 16–87]). Stratified by implantation era, follow-up differed substantially (median 73.5 months for ≤ 2014 vs 15.5 months for ≥ 2019), and incidence rates were 5.46 per 100 patient years (≤ 2014), 8.60 per 100 patient years (2015–2018), and 19.06 per 100 patient years (≥ 2019). For additional context, incidence was 8.52 vs 6.80 per 100 patient years in preterm vs non-preterm patients. Across baseline upright pressure strata, incidence rates were 10.51 per 100 patient years (≤ 20 cmH₂O), 7.53 per 100 patient years (21–25 cmH₂O), and 4.51 per 100 patient years (> 25 cmH₂O). Using a simplified etiology grouping, incidence was 8.52 per 100 patient years for posthemorrhagic hydrocephalus and 15.86 per 100 patient years for spina bifida–associated hydrocephalus, noting wide uncertainty for smaller subgroups.

### Case example

**Fig. 5:**
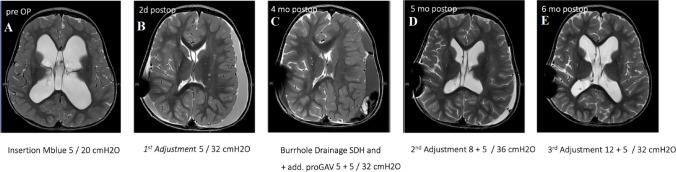
MRI T2-weighted images of a 2-year-old girl taken at different time points, demonstrating ventricular changes and subdural hematoma development over time due to shunt OD and a complex clinical course

A 2-year-old girl with syndromic hydrocephalus presented with accelerated head growth and acquired Chiari type I malformation. MRI T2-weighted sequences revealed enlargement of the ventricular system with evidence of CSF diapedesis. A VP shunt was inserted using an M.blue ventricular valve with a pressure setting of 5/20 cmH₂O. Two days postoperatively, as shown in Fig. 5B, the patient demonstrated small ventricles but developed bilateral SDH. Consequently, the valve setting was adjusted to 5/32 cm H₂O. Serial sonographic monitoring of the ventricular system showed favorable progression. However, at 4 months follow-up, sonography and MRI revealed a slit ventricular system and a new left-sided SDH causing mass effect and midline shift. To address this, burr hole drainage was performed, and a proGAV valve (5 cm H₂O) was added in series with the existing M.blue valve (5/32 cm H₂O) as part of the treatment strategy (Fig. 5C). Continuous sonographic monitoring over the next month demonstrated slight ventricular enlargement with persistent left-sided hygroma. Therefore, the proGAV valve setting was increased to 8 cm H₂O and the M.blue valve to 5/36 cm H₂O (Fig. 5D). At the 6-month follow-up, due to increasing headaches, the proGAV valve setting was adjusted to 12 cm H₂O and the M.blue valve to 5/32 cm H₂O (Fig. 5E). Following these adjustments, the patient remained clinically stable and experienced no shunt-related complications over the subsequent 3 years.

## Discussion

This study provides valuable insights into the outcomes of pediatric patients with hydrocephalus who underwent treatment with GAV ventriculo-peritoneal shunts. After excluding cases with non-standard treatments or missing data, our analysis focused on 223 patients, with a mean follow-up of 5.6 years. This follow-up duration allowed us to observe both immediate and long-term outcomes associated with different shunt configurations. Post-hemorrhagic hydrocephalus was the most prevalent type, accounting for 42% of cases in our cohort, followed by hydrocephalus due to congenital malformations and tumor-related causes. This demographic distribution highlights the diverse etiologies of hydrocephalus in pediatric patients, which may influence treatment outcomes based on the underlying pathology. Our findings reveal that OD was a significant concern in this patient population, affecting 35% of patients. Notably, the occurrence of OD was managed in most cases (81%) through non-surgical adjustments of pressure settings, indicating the importance of programmable valve functionality in reducing the need for surgical intervention. However, 19% of patients with OD (7% of the total patient cohort) required additional surgeries, highlighting the complexity of OD management in certain cases. Similarly, the low mean of 0.3 valve replacement surgeries suggests that the valves themselves are generally reliable; however, the range of up to five replacements in certain cases points to a subset of patients who face recurring complications. Haberl et al. reported in his study, which included 169 patients treated with a wide range of different opening pressures for the paediGAV (between 4/14 and 9/29 cmH₂O), an overall OD rate of 4.7%, a revision rate of 38%, valve obstruction in 4.7% of cases, and proximal catheter obstructions in 10% over a 2-year follow-up [17]. In contrast to Rekate et al., radiological OD in patients with GAV valves can range from less than 10% to as high as 85% in patients followed for more than 5 years [18]. Ros et al., in a series of 166 shunted patients with a mean follow-up of 93 months, found that 56% developed some form of symptomatic OD. The most common reason for surgical revision was valve malfunction [1]. As previously mentioned, the lack of standardized clinical criteria for diagnosis explains the varying rates of shunt OD. In our analyses, we used strict criteria for diagnosing OD, based not only on radiological findings but also on clinical symptoms alone. Pedersen et al., after a systematic review, confirmed that there is currently no clear definition or diagnostic criteria for OD. They suggested that OD in pediatric patients should be defined as a “persistent condition with clinical manifestations such as postural-dependent headache and vomiting, mood changes/irritability, sunken fontanelle, and decreasing head circumference, and/or radiological signs such as slim ventricles/complete ventricular collapse, subdural hygroma/hematoma, and overriding or fused cranial sutures [4].”

When examining the impact of different valve types, the proGAV valve showed a lower OD incidence (15%) compared to the M.blue (36%) and miniNAV/proSA (39%) valves. The Kaplan–Meier curves illustrate a clear and clinically relevant divergence in OD-adjustment–free survival between valve systems. The proGAV valve exhibited the longest adjustment-free durability, which may reflect effective integration of differential pressure and anti-siphon/gravity components that stabilize intracranial pressure across postural changes. Additionally, only 8% of patients with the miniNAV/proSA valve and 7% with the M.blue valve required further interventions, while none of the proGAV patients needed such interventions, further reinforcing the effectiveness of the proGAV valve in this context. The average time to revision surgery due to OD was 72 months, underscoring that while some patients experienced early issues, others remained stable for years before requiring further intervention. The mean initial and latest pressure settings for DPV, GAV, and upright pressure showed an increase in pressure settings for GAV and upright pressure, while the DPV setting remained relatively stable. This trend suggests that increased pressure requirements may arise as patients grow or as their conditions change, particularly for GAV and upright pressure, to better accommodate evolving patient needs over the follow-up period. These findings underscore one more time the importance of programmable valves in the treatment of hydrocephalus, as they allow for adjustments to meet changing patient requirements.

## Strengths and limitations

This study, which includes 223 patients, represents one of the largest cohorts analyzed in the context of OD, thereby strengthening the reliability and generalizability of the findings. However, several limitations should be acknowledged. The retrospective study design may introduce inherent biases, including selection and information bias, which could influence the results. Additionally, due to the substantial heterogeneity in the underlying etiologies of hydrocephalus across valve groups and the resulting imbalance in potential confounders, we were unable to perform further adjusted analyses.

## Conclusion

Long-term follow-up monitoring of shunt-treated pediatric patients reveals a not negligible incidence of OD even though the shunt system is already equipped with a preventive gravitational unit. Pressure level adjustments can effectively counteract corresponding clinical symptoms and radiographic signs. The proGAV valve demonstrated significantly longer adjustment-free survival with lower rates of OD-related interventions, indicating a potential advantage in reducing long-term complications.

## Data Availability

The datasets used and/or analyzed during the current study are available from the corresponding author on reasonable request.

## Abbreviations

*OD*: Overdrainage
*CSF*: Cerebrospinal fluid
*VPS*: Ventriculo-peritoneal shunts
*proGAV*: Programmable gravitational adjustment valve
*MRI*: Magnetic resonance imaging
*miniNAV*: Miniaturized non-adjustable valve
*proSA*: Programmable shunt assistant
*DPV*: Differential pressure valve
*SDH*: Subdural hematoma
